# Sonazoid-enhanced ultrasonography and pathologic characters of CD68 positive cell in primary hepatic perivascular epithelioid cell tumors: A case report and literature review

**DOI:** 10.1515/med-2021-0275

**Published:** 2021-05-11

**Authors:** Chen Li, Jing-Yong Xu, Yuan Liu

**Affiliations:** Department of Ultrasonography, Beijing Hospital, National Centre of Gerontology, Institute of Geriatric Medicine, Chinese Academy of Medical Sciences, Beijing 100730, China; Department of General Surgery, Beijing Hospital, National Centre of Gerontology, Institute of Geriatric Medicine, Chinese Academy of Medical Sciences, No. 1, Dahua Road, Dongdan, Beijing 100730, China

**Keywords:** Sonazoid, CD68, perivascular epithelioid cell tumor, enhanced ultrasonography, Kupffer cell

## Abstract

Perivascular epithelioid cell tumor (PEComa) is a mesenchymal tumor rarely described in the liver. Sonazoid is a new ultrasound contrast with both vascular and post-vascular phases due to the uptake of Kupffer cell. CD68 is a defined immunohistorical staining marker for macrophage including Kupffer cell. No previous cases have been reported to reveal Kupffer images in the post-vascular phase by using Sonazoid and pathologic characters of CD68 positive cell in PEComa. Herein, we describe the first case to present Sonazoid contrast-enhanced ultrasonography (CEUS) findings in Kupffer images and CD68 positive cell in hepatic PEComa which may lead to rethink of the phagocytic properties of macrophages.

## 1 Introduction

Perivascular epithelioid cell tumors (PEComas) are mesenchymal tumors with unique perivascular cells found on the histopathology and immunohistochemistry of liver tissue sections [1]. The PEComa family is comprised of angiomyolipomas, lymphangioleiomyomas, and a group of immunohistochemically similar rare tumors arising at various soft tissues and visceral sites often termed as “PEComas-NOS (not otherwise specified)” [2,3,4]. Hepatic PEComas are rare and challenging to diagnose preoperatively because of nonspecific radiologic features.

Contrast-enhanced ultrasonography (CEUS) using Sonazoid, a new-generation ultrasound contrast agent, can provide the “hemodynamic phase” (or “vascular phase”) like other conventional contrast agents. It detected liver masses by the generation of functional images that allow the radiologist to visualize Kupffer cell distributions, defined as “Kupffer-phase” or “post-vascular phase” [5,6]. Only one patient with PEComa, evaluated with Sonazoid CEUS, has been published [7]; however, the Kupffer-phase was not evaluated in that study. Therefore, in this report, we present the novel imaging characteristics of hepatic PEComa using Sonozoid CEUS, including Kupffer-phase characteristics. This imaging was compared with histopathologic examinations and CD68 immunohistochemical staining characteristics of macrophages and Kupffer cells in liver and tumor tissue sections.

## 2 Case report

We have received informed consent from the patient. A 36-year-old female patient diagnosed with a liver tumor was admitted. The patient was asymptomatic and had no medical history of heavy drinking, oral contraceptives use, hepatitis, or autoimmune disorders. No abnormalities were found on the physical examination or hepatic laboratory functional tests. Serum alpha-fetoprotein, carcinoembryonic antigen, and carbohydrate antigen 19-9 (CA19-9) levels were all within normal intervals.

**Figure 1:**
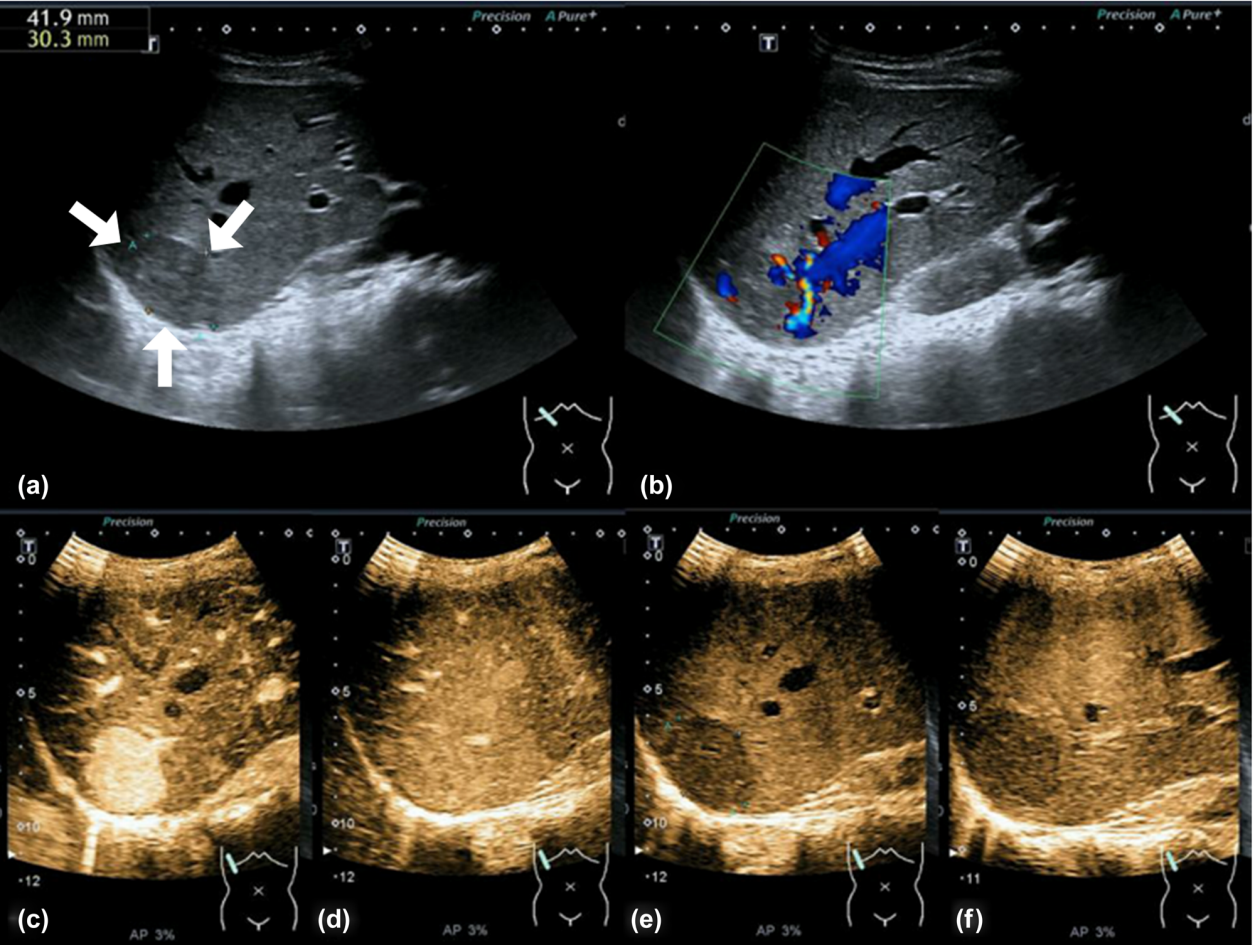
Abdominal ultrasonography and Sonazoid contrast-enhanced ultrasound images (Sonazoid 0.5 mL bolus injection, Toshiba Aplio 500, and 3.75 MHz convex array probe) of a primary hepatic perivascular epithelioid cell tumor. (a) A heterogeneous hypoechoic nodule is demonstrated on B-mode ultrasound (arrows). (b) Color Doppler flow images reveal abundant blood flow at the tumor margins. (c–f) Sonazoid contrast-enhanced ultrasound in the arterial (c, 23 s), portal (d, 100 s), equilibrium (e, 5 min), and post-vascular (f, 10 min) phases. Hyper-enhancement is observed in the arterial phase, with iso-enhancement in the portal phase and mild hypo-enhancement in the equilibrium and post-vascular phases.

**Figure 2:**
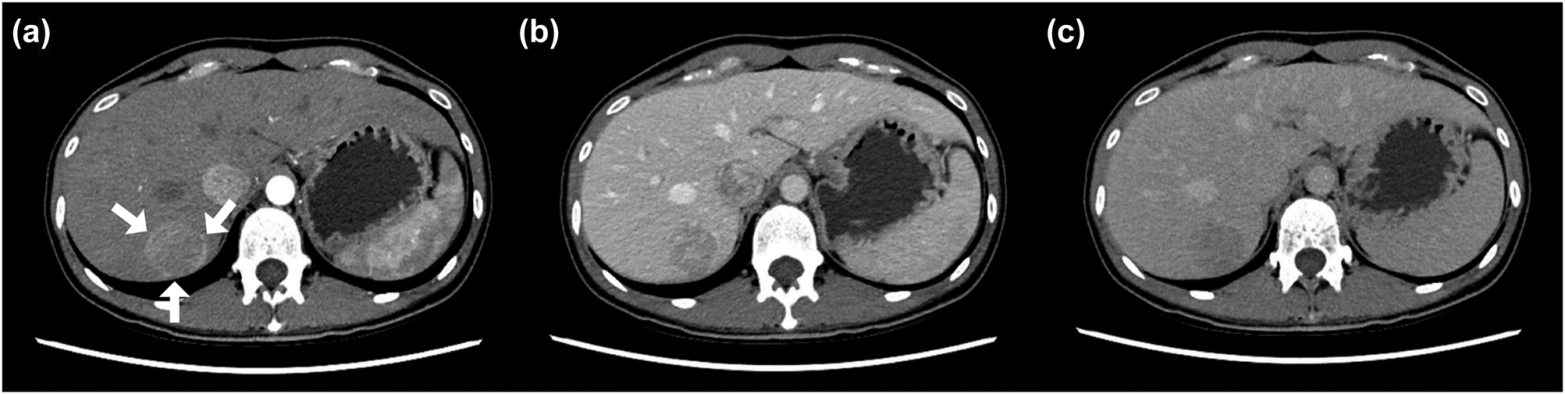
Contrast-enhanced computed tomographic (CT) imaging of a primary hepatic perivascular epithelioid cell tumor in the arterial (a, 25–30 s), portal (b, 60 s), and equilibrium (c, 180 s) phases. A heterogeneous enhancement can be seen during the arterial phase, while the contrast agent was washed out during the portal and equilibrium phases (arrows).

**Figure 3:**
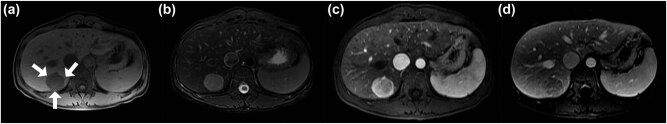
Transverse abdominal MRI using different contrasts, including T1-weighted of a primary hepatic perivascular epithelioid cell tumor (a), T2-weighted (b), and dynamic contrast-enhanced magnetic resonance imaging (c and d). The tumor showed low signal intensity on T1-weighted imaging and high signal intensity on T2-weighted imaging (arrows). Hyper-intensity was seen in the arterial phase (25–30 s), while hypo-intensity was seen in the portal phase (60 s).

Abdominal ultrasonography revealed a heterogeneous hypoechoic mass in the right lobe of the liver, with well-circumscribed margins (Figure 1a). Color Doppler flow images showed blood flow at the margin of the mass (Figure 1b). Sonazoid CEUS (Figure 1c–f) revealed homogeneous hyper-enhancement in the arterial phase, iso-enhancement in the portal phase, and mild hypo-enhancement in the equilibrium phase. Heterogeneous hypo-enhancement was seen in the post-vascular phase. Transverse abdominal contrast-enhanced computed tomography imaging (Figure 2) demonstrated heterogeneous enhancement during the arterial phase and slightly washout during the portal and equilibrium phases. Traverse abdominal magnetic resonance imaging (MRI) of the mass showed low signal intensities on T1-weighted (T1w, Figure 3a) imaging and high signal intensities on T2-weighted (T2w, Figure 3b) imaging. Dynamic contrast-enhanced MRI showed tumor hyper-intensities in arterial phase images (Figure 3c) and hypo-intensities in the portal phase (Figure 3d).

Based on these imaging findings and the medical history, the primary tumor was diagnosed as a hepatic adenoma or atypical hepatocellular carcinoma (HCC).

**Figure 4:**
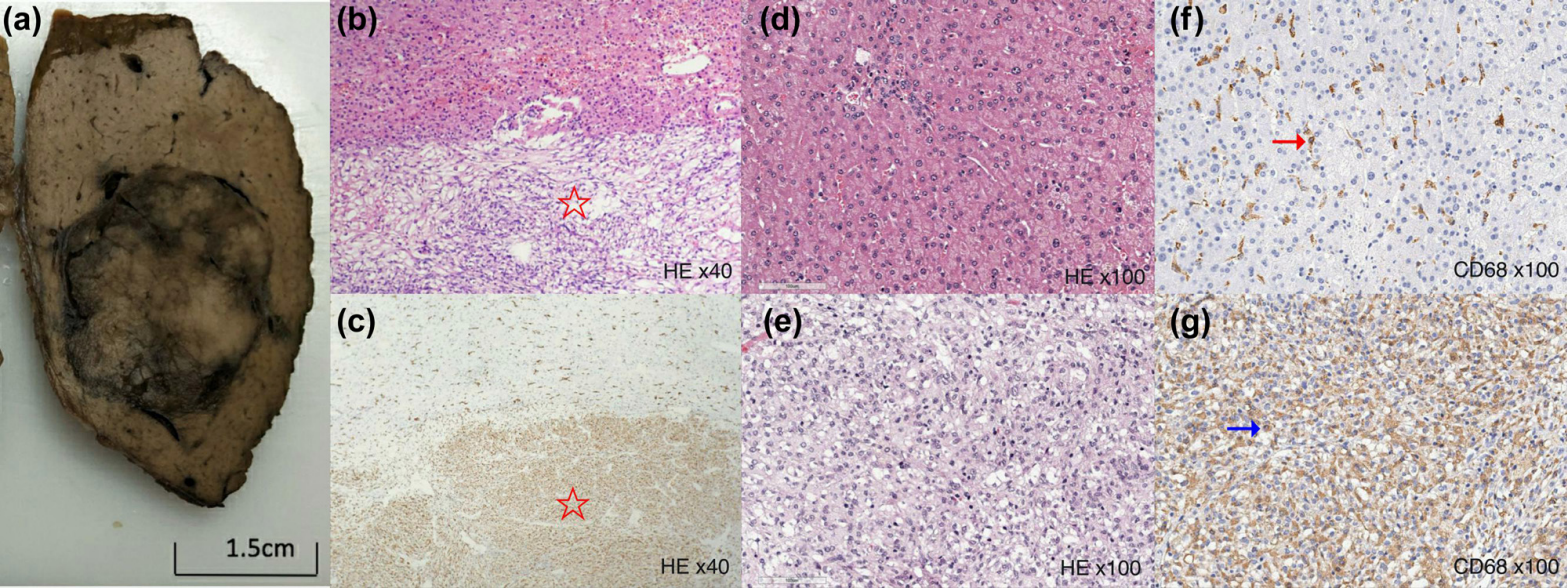
Pathologic and histopathologic findings of a primary hepatic perivascular epithelioid cell tumor. (a) The gross appearance of the tumor on cut dissection shows a well-demarcated brown to gray tumor surrounded by liver tissue. (b) A low power photomicrograph of liver and tumor histopathology using H & E staining (H & E staining ×40). A sharp demarcation between the tumor margin and normal liver parenchyma (star) is observed. (c) A low power photomicrograph of the liver (star) and tumor tissue with CD68 immunohistochemical (IHC) staining (H & E staining ×40); the tumor cells are strongly positive for CD68. (d) and (f) High power photomicrographs of normal liver using H & E and CD68 IHC staining, respectively (H & E staining ×40). (f) Kupffer cells are strongly positive for CD68 and scattered throughout the liver tissue section (arrow). (e) A high power photomicrograph of the tumor with H & E staining (H & E staining ×100). (g) A high power photomicrograph of the tumor using CD68 IHC (H & E staining ×100). CD68 positive cells are densely packed and shaped differently from those of the Kupffer cells in normal liver (arrow).

Resection of liver segment VII was performed. Grossly, the mass was 4.7 × 4 × 3 cm^3^, brown to gray, and well demarcated from the surrounding liver tissue. On microscopic examination, epithelioid and spindle-shaped cells with oval nuclei and clear to granular eosinophilic cytoplasm were mostly seen. Necrosis and nuclear atypia were inconspicuous (Figure 4). Immunohistochemical staining revealed the tumor cells to be positive for smooth muscle actin (SMA), FLI-1, and TFE3, partially positive for human melanin black-45 (HMB-45), and negative for AE1/AE3, ALK, CK8, Desmin, ERG (UMAB78), GPC3, HCC, LCA, Myogenin, S100, and SOX10. The Ki67 proliferative index was 10%. CD68 staining showed strongly positive macrophages but not Kupffer cells in the tumor. The final diagnosis was hepatic PEComa-NOS.

The patient recovered with no postoperative complications and was discharged 1 week after surgery. The evidence of recurrence or metastasis was not found during the follow-up period of 16 months.

## 3 Discussion

In 2013, the World Health Organization (WHO) defined PEComas as mesenchymal tumors composed of distinctive cells showing focal associations with blood vessel walls and usually expressing melanocytic and smooth-muscle markers [8]. Hepatic PEComas are rare and occur primarily in adults with wide age ranges (10–86 years). These tumors are much more frequent in females than males (female-to-male ratio, 2:1 to 5:1) [4]. Most patients are asymptomatic or have no specific clinical symptoms, and the tumors are found incidentally in physical examination. Although the majority of reported PEComas have behaved in a benign fashion, minority have demonstrated malignant behavior with locally destructive recurrence and distant metastasis [9]. However, several other cases can probably exhibit local recurrence or metastasis in long-term follow-up. On histopathology, PEComas are characterized by perivascular locations, and the cells are radially arranged around vascular lumens. Typically, cells around the vessels are epithelioid and spindle-shaped, resembling smooth muscles cells with abundant clear to eosinophilic granular cytoplasm [7,10]. A diagnosis of PEComa usually depends on histopathology and immunohistochemistry [8,11], not on initial diagnostic imaging. PEComas are generally characterized by co-expression of melanocytic markers (HMB-45 and/or Melan-A) and muscle markers (actin and/or desmin) [10]. The tumor, in this case, stained strongly positive for Melan-A, and weakly and partially positive for HMB-45 and SMA, a staining pattern that was key in making a PEComa diagnosis.

The typical ultrasonographic appearance of hepatic PEComa is a well-defined round lesion that is hyperechoic in up to 90% of the cases with high vascularization [12]. Only three liver PEComa cases using CEUS imaging have been reported in English journals [7,13,14]. Sonazoid has been used as a second-generation ultrasound contrast medium in only one case; however, Kupffer imaging in the post-vascular phase has not been reported [7]. SonoVue has been used as another contrast agent in two cases [13,14]. Using SonoVue and Sonazoid contrast agents, homogeneous hyper-enhancements have been obtained in the arterial phase, and iso-enhancement has been seen in the portal vein phase reflected against the surrounding parenchyma. The enhancement patterns in the equilibrium phases were different in PEComas using these two contrast agents; hypo-enhancement was seen using the Sonazoid agent, and persistent slight hyper-enhancement with a lack of rapid washout was observed using the SonoVue agent [7,13,14]. In non-cirrhotic livers, these features can be found in benign lesions such as focal nodular hyperplasia or adenomas, according to the European Federation of Societies for Ultrasound in Medicine and Biology (EFSUMB) Guidelines [14,15]. Aside from benign lesions, in our case, a differential diagnosis of atypical HCC could not be excluded because of the hypo-enhancement in the equilibrium and post-vascular phases.

Sonazoid is a second-generation sonographic contrast agent initially used in Japan and then licensed in China in 2019. This contrast agent contains lipid-shelled microbubbles that can be easily phagocytosed by Kupffer cells resulting in persistent and stable enhancement periods in hepatic parenchyma. This enhancement is termed the post-vascular or Kupffer phase and begins 10 min after the agent is injected and can last from 1 to 2 h [16]. In this case, tumor hypo-enhancement was seen in the post-vascular phase, which could indicate that fewer Kupffer cells were present in this tumor.

CD68 is expressed in monocyte, macrophage, and Kupffer cell cytoplasm, and can be used to identify Kupffer cells in normal and diseased liver tissue sections [17,18]. We expected that liver tissues containing Kupffer cells or CD68 positive cells would be hyper-enhanced on the Sonazoid CEUS Kupffer or post-vascular phases. However, we saw strongly positive CD68 immunohistochemical staining on the tumor tissue sections, but hypo-enhancement of the tumor in the post-vascular phase on Sonozoid CEUS. CD68 positive cells have been observed in hepatic and renal angiomyolipomas and are relatively common in PEComa family tumors [19,20,21,22]. In our case, it is unclear why so many macrophages were present in the tumor. On high power microscopic examination, a morphologic difference between Kupffer cells in the liver and CD68-positive cells in the tumor was seen. These CD68-positive cells were likely histiocytes, such as migrating macrophages and not Kupffer cells [22]. We speculate that tumor inflammatory responses cause a marked increase in migrating macrophages with decreased functional abilities to uptake the Sonazoid contrast agent. This hypothesis requires further research using Sonazoid CEUS and looking at Kupffer phase images to diagnose hepatic PEComas. Additional research is also needed to study the physiologic mechanisms of PEComas [23].

In conclusion, the PEComa in this study showed hypo-enhancement in the Kupffer or post-vascular phases of Sonazoid CEUS with strongly positive CD68 cell staining on tissue sections. The CD68 cells likely represented migrating macrophages with decreased functional abilities, as opposed to Kupffer cells that would have shown enhanced echogenicity on Sonazoid CEUS. This study provides the first characterization of a PEComa using Sonazoid CEUS, which could be used for the noninvasive diagnosis of PEComas.
